# Comparison of the Transcriptome between Two Cotton Lines of Different Fiber Color and Quality

**DOI:** 10.1371/journal.pone.0112966

**Published:** 2014-11-17

**Authors:** Wenfang Gong, Shoupu He, Jiahuan Tian, Junling Sun, Zhaoe Pan, Yinhua Jia, Gaofei Sun, Xiongming Du

**Affiliations:** 1 State Key Laboratory of Cotton Biology, Institute of Cotton Research, Chinese Academy of Agricultural Sciences, Anyang, China; 2 Department of Computer Science and Information Engineering, Anyang Institute of Technology, Anyang, China; National Key Laboratory of Crop Genetic Improvement, China

## Abstract

To understand the mechanism of fiber development and pigmentation formation, the mRNAs of two cotton lines were sequenced: line Z128 (light brown fiber) was a selected mutant from line Z263 (dark brown fiber). The primary walls of the fiber cell in both Z263 and Z128 contain pigments; more pigments were laid in the lumen of the fiber cell in Z263 compared with that in Z128. However, Z263 contained less cellulose than Z128. A total of 71,895 unigenes were generated: 13,278 (20.26%) unigenes were defined as differentially expressed genes (DEGs) by comparing the library of Z128 with that of Z263; 5,345 (8.16%) unigenes were up-regulated and 7,933 (12.10%) unigenes were down-regulated. qRT-PCR and comparative transcriptional analysis demonstrated that the pigmentation formation in brown cotton fiber was possibly the consequence of an interaction between oxidized tannins and glycosylated anthocyanins. Furthermore, our results showed the pigmentation related genes not only regulated the fiber color but also influenced the fiber quality at the fiber elongation stage (10 DPA). The highly expressed flavonoid gene in the fiber elongation stage could be related to the fiber quality. DEGs analyses also revealed that transcript levels of some fiber development genes (Ca^2+^/CaM, reactive oxygen, ethylene and sucrose phosphate synthase) varied dramatically between these two cotton lines.

## Introduction

Upland cotton (*Gossypium hirsutum* L.) is the largest natural fiber producer of the plants. In recent years, interest in naturally colored cotton has grown because it may reduce pollution, making it preferable to white fiber which requires a dyeing process [1]–[3]. However, its commercial application is very limited due to the lack of fiber color diversity and low fiber quality [2]. Limited brown (different color depth) and green fiber lines, among other varieties, have been used in the textile industry. A previous study demonstrated that there was a significant negative correlation between the degree of fiber color and lint percentage and fiber quality traits in cotton [4]. Therefore, subsequent studies should focus on improving the fiber quality and revealing the underlying mechanisms for pigmentation formation in naturally colored cotton.

Early genetic analysis suggested that the brown color of cotton fiber was controlled by one incompletely dominant major gene [5]. Furthermore, gene expression analysis and dimethylaminocinnaldehyde staining showed that tannins could be the key chemical responsible for the brown color in cotton fiber [6]. Subsequent chemical research indicated that the brown pigment in cotton fiber might be the chinone compound oxidated from condensed tannins, and the accumulation period of condensed tannins was from 10 DPA-25 DPA [7], [8]. However, the molecular mechanism that underlies pigmentation in colored cotton fiber is still unknown.

With the development of next generation sequencing technology, RNA-seq provides a powerful tool to rebuild our knowledge of transcriptomics. By directly sequencing and assembling the mRNA, the whole transcriptome could be de novo reconstructed precisely and efficiently [9], aligned with public databases for function annotation and the critical genes could be assessed using pathway classification. In addition, gene expression can be measured and the number of transcripts can be obtained if the appropriate level of sequencing was performed. The application of RNA-seq technology has been used successfully for various species [10]–[14].

Lines Z263 and Z128, two brown fiber inbred lines with dark and light brown fiber respectively, both derived from a cross between white and brown fiber cotton. To reveal the whole transcriptome landscape of the natural colored cotton fiber, and to understand the molecular mechanism of pigment formation, the transcriptomes of these two lines at the whole early developmental stage (0 dpa–20 dpa) were sequenced using RNA-seq technology and analyzed.

## Materials and Methods

### 1. Plant material

Line Z263 (*Gossypium hirsutum* L.), with dark brown fiber, was selected from a cross between white cotton (Zhong 6331) and brown fiber cotton (Crd). Line Z128 (*G. hirsutum* L.) was a selected mutant from Z263. They were planted in an experimental field at the Institute of Cotton Research, Chinese Academy of Agricultural Sciences (ICR, CAAS) under normal agronomic management conditions. The bolls at the day of anthesis (0 day post anthesis/DPA), 5 DPA, 15 DPA and 20 DPA were harvested and stored in an ice box, then the ovules of 0 DPA and fibers of other stages were dissected and separated on ice as fast as possible, and then stored at −80°C immediately.

### 2. Fiber quality measurement, fiber microstructure detection and cellulose test

To test the fiber quality of Z263 and Z128, the following measurements were included: upper half mean length (mm), uniformity index (%), micronaire, fiber elongation (%), fiber strength, 15 g lint samples of each line were analyzed using USTER HVI 1000 (USTER Technologies, Inc., Uster, Switzerland). The fiber microstructure detection and cellulose test were performed according to Ru et al [15].

### 3. RNA extraction and cDNA library construction

Total RNAs were extracted from each sample using the CTAB method described by Wan and Wilkins [16], with minor modifications to increase the yield. All RNA quality and quantity were measured by 1.0% agarose gel and an ultraviolet spectrophotometer. Four stages of RNAs (0 DPA, 5 DPA, 15 DPA and 20 DPA) with the same concentration and quality from each line were mixed together as one mixed library. The two libraries of Z263 and Z128 were constructed using the method described by Xia et al. [17].

### 4. RNA-seq and sequence de novo assembly

The sequencing of two libraries of Z263 and Z128 were performed on HiSeq 2000 (illumina) by the Beijing Genomics Institute (BGI) (Shenzhen, Guangdong, China). The raw reads, transformed from images, were first processed by removing adaptors and redundant fragments to generate clean reads. The clean reads de novo assembly was carried out using the short reads assembling program-SOAPdenovo [18]. Clean reads with a certain length of overlap were first combined to form “contigs”. Then the clean reads were mapped back to the contigs; it was possible to detect contigs from the same transcript, as well as the distances between them, by paired-end reads. Next, SOAPdenovo connected the contigs into “scaffolds”; “N” was used to present unknown sequences. Paired-end reads were used again to fill the gaps between scaffolds, longer sequences which could not extend at either end were assembled and defined as “unigenes”. In this study, the unigenes from the two libraries were further processed by sequence splicing and redundancy removal with the sequence cluster program-TGICL [19] to acquire non-redundant unigenes that were as long as possible.

### 5. Unigene functional annotation and functional categorization

All-unigenes were first searched using the blastx tool against public protein databases such as Non-redundant protein sequences (nr, http://www.ncbi.nlm.nih.gov), Swiss-Prot (http://www.expasy.org/sprot/), Kyoto Encyclopedia of Genes and Genomes (KEGG, http://www.genome.jp/kegg/pathway.html) and Cluster of orthologous groups for eukaryotic complete genomes (KOG, http://genome.jgi.d oe.gov/Tutorial/tutorial/kog.html). An e-value<10^−5^ was used as the threshold. To further understand the distribution of gene function, the protein functional classification and pathway were annotated by Gene Ontology (http://www.geneontology.org/), KOGs and KEGG. With NR annotation, GO functional annotation and classification were obtained using the Blast2GO program [20] and WEGO software [21], respectively.

### 6. Differential expressed genes (DEGs) identification and enrichment analysis

Referring to the method described by Audic and Claverie [22], the Beijing Genomics Institute (BGI) developed a rigorous algorithm to identify DEGs from two samples of RNA-seq data. Because the expression of each gene occupies only a small part of the library, we denote the number of unambiguous clean tags from gene A as x, and the p(x) is in the Poisson distribution. The formula is as follows:
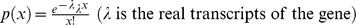



The total clean tag number of sample 1 is N^1^, and total clean tag number of sample 2 is N^2^; gene A holds x tags in sample 1 and y tags in sample 2. The probability of gene A expressed equally between two samples can be calculated with the following formula:
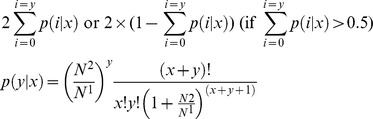



The P value corresponds to the differential gene expression test.

The false discovery rate (FDR) is a method used to determine the threshold of the P value in multiple tests. In our study, the reads per kilobase of exon model per million mapped reads (RPKM) value, was calculated referring to the formula described by Mortazavi et al. [23] and was used to quantify the transcript level of Z128 versus Z263. FDR≤10^−3^ and the absolute value of the log2Ratio (Z128_RPKM/Z263_RPKM) ≥1 as the threshold, were used to judge significant differences in gene expression. DEGs were then subjected to GO functional enrichment analysis and KEGG pathway enrichment analysis.

GO functional enrichment analysis provides GO terms, which significantly enrich in DEGs compared to the genome background, indicating that the DEGs are connected to interesting biological functions. All DEGs are firstly mapped to GO terms in the database, calculating gene numbers for every term, then the ultra-geometric test is used to find significantly enriched GO terms in DEGs compared to the genome background. The formula is:
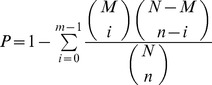



Where N is the number of genes with GO annotation; n is the number of DEGs in N; M is the number of genes that are annotated according to the GO terms; m is the number of DEGs in M. The calculated P value≤0.05 was taken as a threshold. GO terms fulfilling this condition are defined as significantly enriched GO terms in DEGs. This analysis recognizes the main biological functions that DEGs participate in.

Pathway enrichment analysis identifies significantly enriched metabolic pathways or signal transduction pathways in DEGs compared with the whole genome background. The formula is the same as that in GO analysis. Here, N is the number of genes with KEGG annotation, n is the number of DEGs in N, M is the number of genes related to specific pathways, and m is number of DEGs in M (Qvalue≤0.05). All DEGs are further mapped on to each pathway; up-regulated genes are marked with red borders while down-regulated genes are marked with green borders.

### 7. qRT-PCR analysis for genes related to flavonoid synthesis

The genes and primers used for the gene expression analysis related to flavonoid synthesis were listed in Table 1. qRT-PCR was performed in a total volume of 20 µL with 10 µL SYBR Premix Ex Taq(2×) (Takara, Japan), PCR forward primer (10 µM) 0.4 µL,PCR reversed primer 0.4 µL (10 µM),ROX Reference Dye II (50×) 0.4 µL,cDNA template 2.0 µL and ddH_2_O 6.8 µl on a 7500 real-time PCR machine (Applied Biosystems) according to the manufacturer’s instructions. PCR amplification employed a 10 s denaturing step at 95°C, followed by 5 s at 95°C and 40 s at 60°C with 40 cycles. Relative mRNA levels were calculated by the 2^−ΔΔCT^ method with *Gh18S* (accession number: L24145) as an internal control.

**Table 1:** The primers used in this study.

Genename	NCBIaccession no.	Direction	PrimerSequence (5′→3′)	Productlength
CHS	EF643507	F	GGTGTGGACATGCCTGGGGC	265
		R	CAGCTGCGGCACCATCACCA	
CHI	EF187439	F	ATCCGTTGAGTTTTTCAGAG	127
		R	CCAAATAGCAACGCAAT	
F3′H		F	CGAGGAGATGGATAAGGTGATTG	128
		R	GCAAGTTCAAGGGAGTAGATGGA	
F3H	EF187440	F	GCTTCTTGAGGTGTTGTCAGAGG	116
		R	CAGGTTGAGGGCATTTAGGATAG	
DFR	FJ713480	F	CGCGACCCTGGCAACTCGAA	417
		R	CCAGGCTGCTTGCTCTGCCA	
ANS	EU921264	F	AAGAGAAGTATGCCAACGAC	102
		R	AGAAGTAGTCCTCCCACTCA	
ANR	FJ713479	F	TCCTCAACAAAAGATACCCTGACTT	147
		R	CGGTTTGGTCGTAGATTTCCTC	
LAR		F	AAAGTAGCCAAAGCCCTTCA	260
		R	TAACAGTGCCGACAGAGTGAA	
Gh18S	L24145	F	TGACGGAGAATTAGGGTTCGA	100
		R	CCGTGTCAGGATTGGGTAATTT	

### 8. Statistical analysis

All of the experiments concerning data comparisons were performed three times. Statistical analyses were performed using the S-N-K method of independent-samples t-test at 95% confidence with IBM SPSS Statistics 11.0 (SPSS Inc., Chicago, USA). Values with different lowercases represent a significant difference at P<0.05.

## Results

### 1. The differences in fiber color and quality between Z263 and Z128

**Figure 1:**
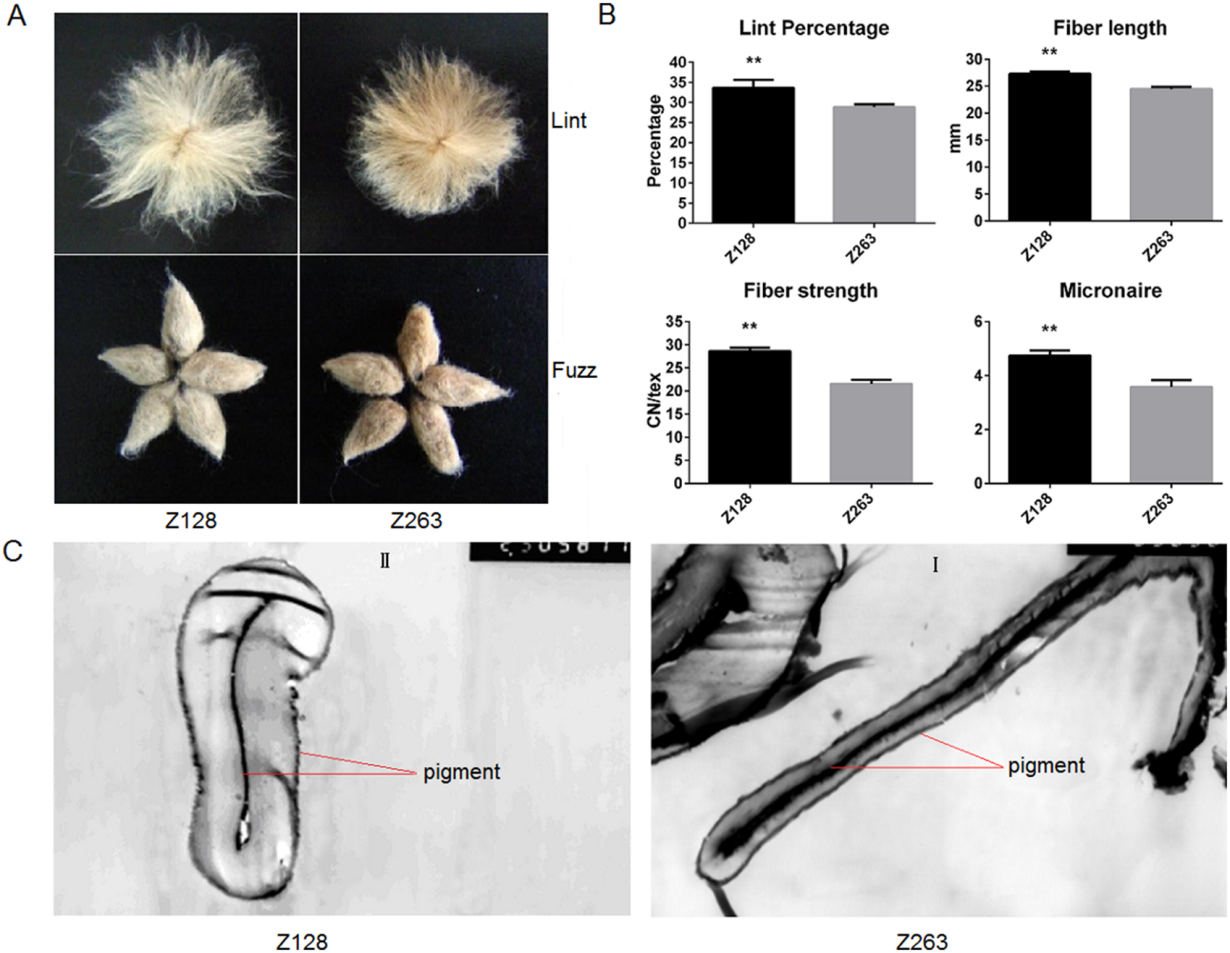
The fiber color, yield, quality and microstructure of Z128 and Z263. Statistical analyses were performed at 95% condence with IBM SPSS Statistics 11.0 (SPSS Inc., Chicago, USA). Values with an asterisk represented a significant differe- nce at P<0.05. The fiber microstructure of Z263 (C I) and Z128 (C II) was observed under a microscope (×3000 and ×2500, respectively).

Line Z128 was a selected mutant from Z263 and both lines have similar genetic backgrounds. However, their fibers were different. As shown in Fig. 1, the fiber of Z263 was dark brown while that of Z128 was light. Though the primary walls of the fiber cell in both Z263 and Z128 contain pigments (Fig. 1), more pigments were laid in the lumen of the fiber cell in Z263 compared with that Z128. This resulted in a darker color in the Z263 fiber. However, the fiber yield and quality of Z128 was better than that of Z263. The lint percentages (%), upper half mean length (mm), micronaire and fiber strength in Z128 were also better than those in Z263 (Fig. 1). Furthermore, Z128 contained more cellulose (98.5%) than Z263 (94.5%).

### 2. The de novo assembled transcriptome of the fibers in Z263 and Z128

By removing useless sequences, a total of 38,114,054 (2,858,554,050 nucleotides) and 39,355,642 (2,951,673,150 nucleotides) 75 bp-length clean reads were obtained from the Z128 and Z263 mRNA libraries, respectively. A total of 170,201 and 182,404 contigs, 143,588 and 151,478 scaffolds and 59,926 and 71,895 unigenes were assembled in the Z128 and Z263 library, respectively. Because both of the samples for library construction were collected from the same tissue (ovule and fiber), two unigene libraries were taken forward for sequence clustering and redundancy removal to generate a new unigene library (All-unigene library) to make the non-redundant unigenes as long as possible. The All-unigene library contained 71,895 unigenes with an average length of 533 bp, which was obviously greater and longer than the other two libraries. The length of most of the unigenes in the three libraries was in the range of 100–1,000 bp, accounting for 93.08%, 92.44% and 88.21% in Z128-, Z263- and all-unigene library, respectively.

Since the all-unigene library contained the most complete and longest sequences, it was used to run batch alignment with a cut-off E-value of 10^−5^ on online public databases. A total of 49,941 (69.46%) and 31,714 (44.11%) unigenes received annotations from the NCBI non-redundant (nr) and Swiss-Prot databases, respectively. KEGG, KOGs and GO similarity analyses indicated that 20,241 (28.15%), 14,333 (19.94%) and 7,757 (10.79%) unigenes matched these databases, respectively. To investigate the genomic similarity between *Gossypium* and other species, we estimated all annotations of unigenes from nr. The result showed that the most abundant unigenes were annotated as “*Vitis vinifera*”, “*Ricinus communis*” and “*Populus trichocarpa*”, which accounted for 29.71%, 29.59% and 24.63%, respectively. Furthermore, we annotated 68.2% and 80.2% unigenes on the recently r- eleased A and D genome of diploid cotton (which were considered to be two donor g- enomes of the tetroploid cotton subgenome), respectively.

**Figure 2:**
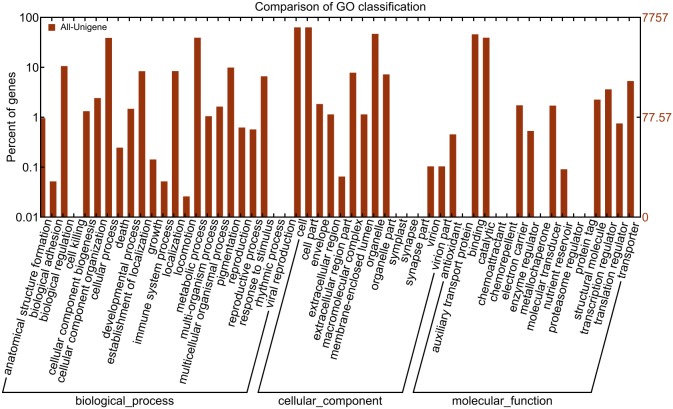
All-unigenes classified by GO analysis.

GO classification analysis showed that 7,757 all-unigenes were categorized into three main ontologies: biological process, cellular component and molecular function, which were further categorized as 54 terms, and all-unigenes were classified into different terms. One unigene might be repeatedly classified in different terms, therefore, a total of 32,935 all-unigenes (including 75 unigenes repeated in different categories) were distributed over 42 terms, 14,867 (45.14%) of them were categorized in cellular component ontology, 10,229 (31.06%) in biological process and 7,839 (23.80%) in molecular function (Fig. 2). The detailed classification demonstrated that the term “cell” (4,876) and “cell part” (4,876) in “cellular component” ontology, “metabolic process” (3,026) and “cellular process” (3,002) in “biological process” ontology, “binding” (3,538) and “catalytic” (3,030) in “molecular function” contained the most unigenes, respectively. Furthermore, 764 unigenes under the term “pigmentation” indicated that an abundance of pigment-related biological processes were involved in the development of colored cotton fiber.

**Figure 3:**
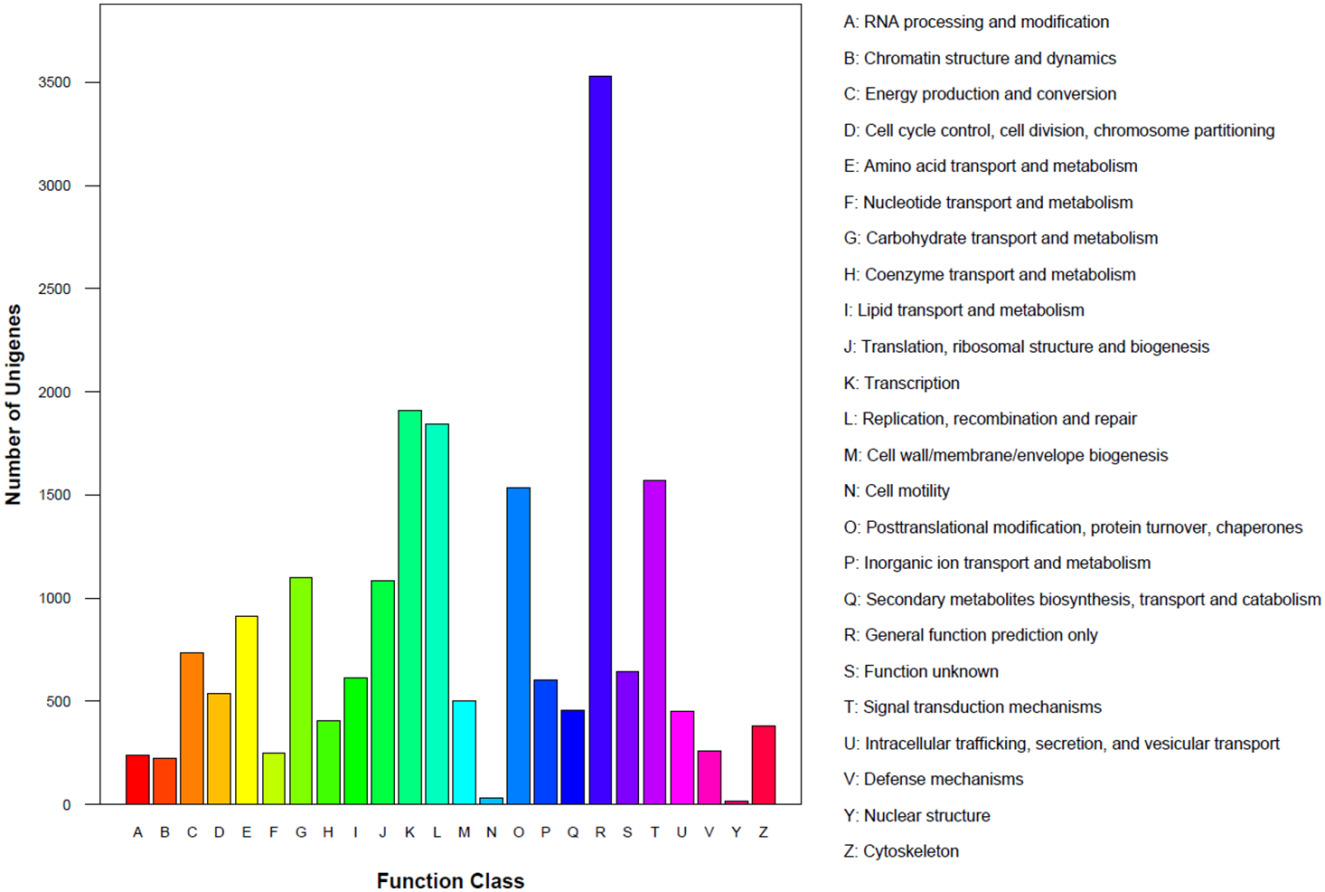
COG classification of all-unigenes. A–Z represented different functions classified by GO analysis, respectively.

The all-unigenes library was aligned to the KOG database to predict and classify possible functions. A total of 14,333 unigenes were matched and categorized into 24 classes (Fig. 3). The function class defined as “general function prediction” (code: R) had the most unigenes (24.61%), followed by “transcription” (K: 13.33%), “replication, recombination and repair” (L: 12.86%), “signal transduction mechanisms” (T: 10.95%) and “posttranslational modification, protein turnover, chaperones” (O: 10.70%).

The all-unigene library was aligned with the KEGG pathway database, and the result showed a total of 38,645 unigenes which were classified into six categories, mostly concentrated in three of them: metabolism (11,371; 29.42%), protein families (10,638; 27.53%) and cellular processes (6,959; 18.01%). Furthermore, 1,315 unigenes were clustered in the “biosynthesis of secondary metabolites” category (Table S1).

### 3. Differentially expressed genes (DEGs) analysis

**Figure 4:**
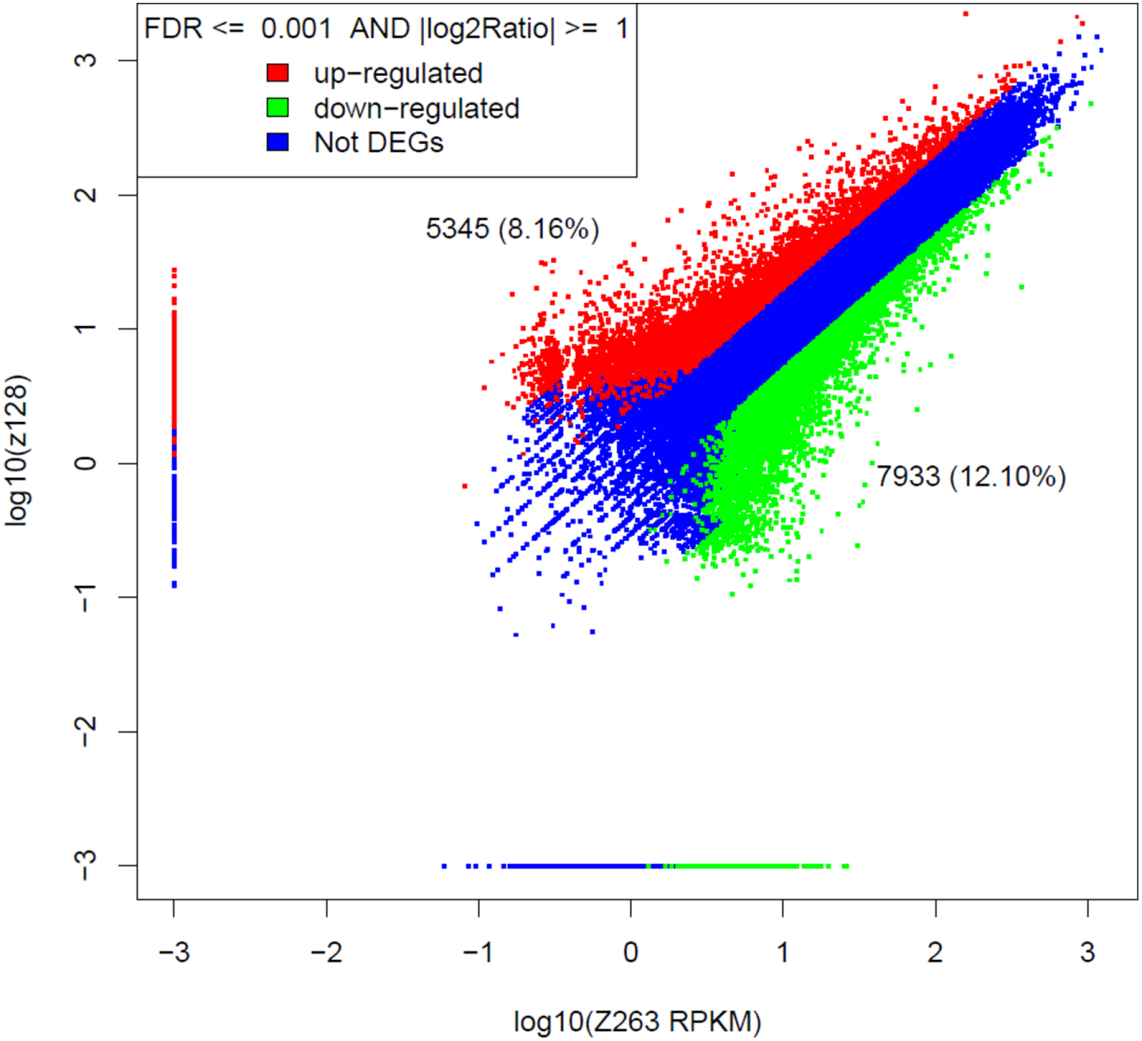
Mapping of all expressed unigenes. This figure was created by comparing gene expression levels of Z128 to Z263. FDR≤0.001, |log_2_Ratio| ≥1 was used as threshold, red dots represented up-regulated genes, green dots represented the down-regulated genes, and blue dots represented gene expression with no significant difference.

The unigene expression level in the Z128 and Z263 libraries were compared. A total of 13,278 (20.26%) unigenes were significantly differentially expressed when these two non-redundant libraries were compared (FDR≤0.001, |log_2_Ratio|≥1). A total of 5,345 (8.16%) of them were up-regulated, while 7,933 (12.10%) of them were down-regulated; the others were not DEGs (Fig. 4).

The GO analysis results showed that when corrected (P-value≤1), seven enriched terms belonged to three ontologies, one of them was categorized in “biological process”, three were in “cellular component” and three were in “molecular function” (Table 2).

**Table 2:** The DEGs enriched terms in GO analysis (P-value<1).

Ontology	Gene Ontology term	Clusterfrequency	GenomeFrequencyof use	CorrectedP-value
Biologicalprocess	RNA-dependent DNA replication	15/597, 2.5%	47/4110, 1.1%	0.82163
Cellularcomponent	cytoplasmic vesicle	137/696, 19.7%	750/4911, 15.3%	0.06344
	cytoplasmicmembrane-bounded vesicle	135/696, 19.4%	745/4911, 15.2%	0.10093
	spliceosome	7/696, 1.0%	14/4911, 0.3%	0.23912
Molecularfunction	oxidoreductase activity, actingon paired donors, withincorporation or reductionof molecular oxygen,2-oxoglutarate as one donor,and incorporation of oneatom each of oxygen intoboth donors	7/769, 0.9%	13/5273, 0.2%	0.32203
	RNA-directed DNApolymerase activity	15/769, 2.0%	48/5273, 0.9%	0.76482
	DNA polymerase activity	15/769, 2.0%	49/5273, 0.9%	0.95719

Table 3 showed the top 10 DEGs enriched pathways, seven of which were categorized as a “metabolism” pathway, two were categorized as “genetic information processing” pathways and the other one was categorized as “environmental information processing”. Further identification indicated that five of the “metabolism” terms belonged to the “biosynthesis of secondary metabolites” sub-category.

**Table 3:** The top 10 DEGs enriched pathways in KEGG analysis.

No.	Pathway	DEGs withpathwayannotation(3,213)	All genes withpathwayannotation(20,242)	P-value	Pathway ID
1	Zeatinbiosynthesis	16 (0.5%)	41 (0.2%)	0.000299	ko00908
2	Anthocyaninbiosynthesis	4 (0.12%)	5 (0.02%)	0.002767	ko00942
3	ABC transporters	40 (1.24%)	164 (0.81%)	0.003	ko02010
4	Diterpenoidbiosynthesis	15 (0.47%)	47 (0.23%)	0.004717	ko00904
5	Phenylpropanoidbiosynthesis	56 (1.74%)	255 (1.26%)	0.006239	ko00940
6	3-Chloroacrylicacid degradation	18 (0.56%)	63 (0.31%)	0.007641	ko00641
7	Flavone andflavonol biosynthesis	14 (0.44%)	45 (0.22%)	0.007987	ko00944
8	Ubiquitinmediatedproteolysis	108 (3.36%)	550 (2.72%)	0.00975	ko04120
9	Taurine andhypotaurinemetabolism	5 (0.16%)	10 (0.05%)	0.012555	ko00430
10	Base excisionrepair	36 (1.12%)	158 (0.78%)	0.014168	ko03410

### 4. Expression of related genes for color and fiber development in two cotton lines of different fiber color and quality

The pigmentation deposits in the brown colored cotton fiber were closely related to the flavonoid and proanthocyanidins biosynthesis. In this study, the “anthocyanin biosynthesis” and “flavone and flavonol biosynthesis” appeared on the top 10 list of the KEGG enrichment analysis (Table 3). Further analysis using the KEGG database indicated that a total of 14 unigenes were involved in the “flavone and flavonol biosynthesis” pathway (Table 4), which could be further classified as three orthology categories. “K05280” contained one up-regulated unigene which encoded flavonoid 3′-hydroxylase (F3′H). In addition, 10 down-regulated unigenes that encoded flavonol 3-O-methyltransferase (OMT) belonged to “K05279”; in “K10757”, all unigenes encoded flavonol 3-O-glucosyltransferase (FOGT), two of them were up-regulated, and one was down-regulated. A total of four unigenes were mapped in the “anthocyanin biosynthesis” pathway, all of them were up-regulated. All four unigenes were involved in anthocyanin 5-O-glucosyltransferase (5GT) encoding.

**Table 4:** The involved unigenes in Flavone and flavonol biosynthesis and Anthocyanin biosynthesis pathways.

	Orthology	Entry ofenzyme	Unigenes	Ratio^1^	Status^2^	Encoded protein
Flavonoidbiosynthesis	K00660	2.3.1.74	Unigene56280_All	−1.9	D	chalcone synthase (CHS)
	K00588	2.1.1.104	Unigene34142_All	−1.4	D	caffeoyl-CoA O-methyltransferase
			Unigene42690_All	−3.6	D	
			Unigene10308_All	−1.1	D	
	K01859	5.5.1.6	Unigene69406_All	−1.6	D	chalcone isomerase (CHI)
	K05277	1.14.11.19	Unigene56780_All	−2.3	D	anthocyanidin synthase (ANS)
			Unigene57093_All	−3.0	D	
			Unigene5167_All	−1.6	D	
	K08695	1.3.1.77	Unigene43073_All	−1.1	D	anthocyanidin reductase (ANR)
	K05280	1.14.13.21	Unigene71334_All	2.8	U	flavonoid 3'-hydroxylase (F3'H)
	K00475	1.14.11.9	Unigene70267_All	3.9	U	flavanone 3-hydroxylase (F3H)
			Unigene71550_All	4.2	U	
	K05278	1.14.11.23	Unigene70581_All	4.1	U	flavonol synthase (FLS)
			Unigene6472_All	1.0	U	
Flavone andflavonolbiosynthesis	K05280	1.14.13.21	Unigene71334_All	2.8	U	flavonoid 3'-hydroxylase (F3'H)
	K05279	2.1.1.76	Unigene13368_All	−2.2	D	flavonol 3-O-methyltransferase (FOMT)
			Unigene21288_All	−1.4	D	
			Unigene38018_All	−1.2	D	
			Unigene47446_All	−2.4	D	
			Unigene47563_All	−3.7	D	
			Unigene50636_All	−2.7	D	
			Unigene57809_All	−1.7	D	
			Unigene58594_All	−1.9	D	
			Unigene70908_All	−1.7	D	
			Unigene9272_All	−1.1	D	
	K10757	2.4.1.91	Unigene50839_All	1.0	U	flavonol 3-O-glucosyltransferase (FOGT)
			Unigene8998_All	2.1	U	
			Unigene54635_All	−4.3	D	
Anthocyaninbiosynthesis	K12338	2.4.1.298	Unigene18122_All	2.2	U	anthocyanin 5-O-glucosyltransferase (5GT)
			Unigene59143_All	12.0	U	
			Unigene67316_All	12.5	U	
			Unigene71660_All	12.9	U	

1: ratio indicated log2(z128_RPKM/Z263_RPKM).

2: “U” indicated that this unigene was up-regulated; “D” indicated down-regulated.

**Figure 5:**
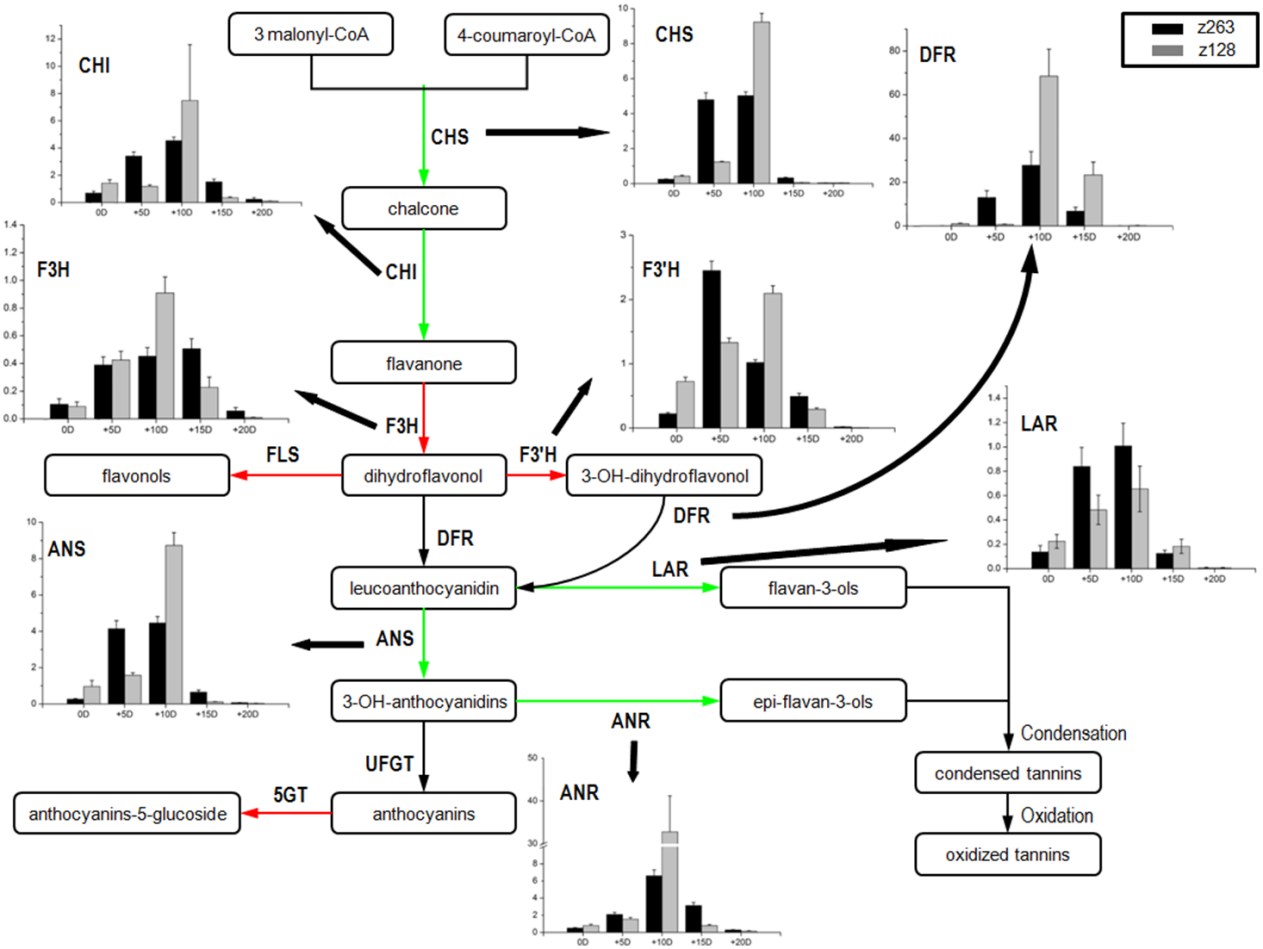
The schematic of pigment formation in cotton fiber. The red and green arrows indicated the up- and down-regulated status detected by comparing gene expression in Z128 with that in Z263. chalcone synthase (CHS); chalcone isomerase (CHI); flavanone 3-hydroxylase (F3H); flavonol synthase (FLS); dihydroflavonol 4-reductase (DFR); leucoanthocyanidin reductase (LAR); anthocyanidin synthase (ANS); UDP-favonoid glucosyl transferase (UFGT); anthocyanidin reductase (ANR); anthocyanin 5-O-glucosyltransferase (5GT).

Another important pigmentation pathway in plants is the “flavonoid biosynthesis pathway”, which is also considered to be a key pathway for pigment formation in brown fiber cotton. Although it was not shown in Table 3, eight important genes were involved in this pathway. Chalcone synthase (*CHS*), chalcone isomerase (*CHI*), leucoanthocyanidin reductase (*LAR*), anthocyanidin reductase (*ANR*) and anthocyanidin synthase (*ANS*) were down-regulated in this pathway, while flavonoid 3′-hydroxylase (*F3′H*), flavanone 3-hydroxylase (*F3H*) and flavonol synthase (*FLS*) were all up-regulated. According to the distribution of DEGs in the entire pathway, most of the down-regulated DEGs (*CHS*, *CHI*, *LAR*, *ANR*, *ANS*) were enriched in upstream and downstream pathways, and the up-regulated DEGs were in the middle of the flavonoid biosynthetic pathway (*F3H*, *F3′H*, *FLS*). However, another gene in the middle of the pathway, the dihydroflavonol 4-reductase (*DFR*), was unchanged (Fig. 5). Furthermore, a gene that encodes 5-O-glucosyltransferase in the anthocyanin biosynthesis pathway was up-regulated. The down-regulated genes in the flavonoid biosynthesis pathway suggested that there were less pigments in Z128 compared with that in Z263. This was confirmed by the lighter brown fiber color in Z128 compared to the dark brown fiber in Z263.

To test the reliability of comparative transcriptional data, qRT- PCR analysis was performed for *CHS*, *CHI*, *LAR*, *DFR*, *F3H*, *F3′H*, *ANR* and *ANS*. Samples were selected from the flavonoid biosynthesis pathway across five developmental time points from 0 DPA to 20 DPA. Overall, the results of the qRT-PCR analysis were consistent with the results from the transcriptome for the mixed mRNAs of five developmental time points of the eight selected genes. However, when Z128 was compared to Z263 at 10 DPA, the selected eight genes had significantly higher transcript levels in Z128 (Fig. 5). This suggests that the genes involved in the flavonoid biosynthesis pathway at 10 DPA are related to better fiber quality formation. Moreover, the genes involved in flavonoid biosynthesis affected the fiber color and fiber quality of the brown fiber cotton. Some other genes such as the ethylene related factors also regulated fiber development [24]. According to Table 5, all of the ethylene related factors had higher expression levels in Z128 than in Z263. The DEGs analysis also revealed that transcript levels of different fiber development related factors varied dramatically in cotton fibers, such as reactive oxygen [25], ethylene [24], Ca^2+^/CaM [26], and sucrose phosphate synthase [27] (Table 5).

**Table 5:** Fiber development related DEGs of brown and white fibers.

genes	Z263 (RPKM)	Z128 (RPKM)	p-value	FDR	Ratio^1^	Status^2^	Homologous proteins
Unigene31293_All	40.4792	19.2683	5.06E-15	2.40E-16	−1.1	D	extracellular Cu/Zn superoxide dismutase
Unigene15009_All	36.5823	3.0274	1.54E-52	2.92E-54	−3.6	D	class III peroxidase
Unigene22859_All	1.8516	5.7002	4.97E-04	1.53E-04	1.6	U	glutathione peroxidase
Unigene25776_All	2.1838	9.8675	1.78E-09	2.04E-10	2.2	U	peroxisomal membrane ABC transporter family
Unigene40537_All	80.13	230.84	2.93E-14	1.51E-15	1.5	U	fiber quinone-oxidoreductase
Unigene50168_All	2.78	1.20	3.83E-02	2.08E-02	−1.2	D	calcium-transportingATPase(ACA9)
Unigene1176_All	16.85	45.17	2.03E-13	1.24E-14	1.4	U	calcium ion transmembrane transporter(ACA2)
Unigene55005_All	3.59	0.50	1.55E-03	5.46E-04	−2.8	D	calcium ion binding/transporter(ATNRT1∶2)
Unigene15884_All	3.32	14.7	2.71E-14	1.38E-15	2.1	U	autoinhibited calcium ATPase
Unigene61435_All	1.1851	5.9315	3.37E-04	1.00E-04	2.3	U	ethylene receptor
Unigene40770_All	5.06	14.98	2.02E-07	3.10E-08	1.5	U	ethylene responsive element binding protein
Unigene26099_All	4.86	61.24	0.00	0.00	3.7	U	ethylene responsive transcription factor 2b
Unigene37372_All	22.708	90.2862	0.00	0.00	1.9	U	ethylene-responsive element binding protein ERF2
Unigene62869_All	0.86	9.38	2.65E-05	6.01E-06	3.4	U	Ethylene-responsive transcription factor 1B
Unigene30365_All	6.59	21.61	6.11E-14	3.33E-15	1.7	U	sucrose phosphate synthase

1: ratio indicated log2(z128_RPKM/Z263_RPKM);

2: “U” indicated that this unigene was up-regulated; “D” indicated down-regulated.

## Discussion

The RNA-seq approach based on next generation sequencing technology provided us with a new method to study the transcriptome of developing cotton fibers. It was not dependent on existing genome information and was an efficient way to quantify the expression level of a single gene without high background noise [28]. In recent years, this technology has been successfully applied in transcriptome studies for many non-model organisms [13], [17], [29]–[30].

In this study, we mixed the RNA from four important fibers at various developmental stages (5, 10, 15 and 20 DPA) to de novo assemble the transcriptome of developmental colored cotton fiber. A total of 125,014 unigenes were generated in two sequencing libraries, which were further assembled into 71,895 all-unigenes with an average length of 533 bp, 69.46% of which could be matched in the NCBI database (E-value≤10^−5^). This volume of data was greater than that reported in previous studies on other species [17], [30]. Approximately 20,000 unigenes identified from *G. hirsutum* were recorded in the NCBI database (Nov 2011). In this study, approximately 50,000 unigenes (EST) were matched with nr database records. Therefore, we believe that our unigene library contained almost all of the known unigenes from *G. hirsutum*. In conclusion, we acquired a high quality and well-assembled transcriptome library for developing colored cotton fibers.

In all nr-annotated unigenes, only 1,537 (3.08%) were directly annotated with the field of “*Gossypium hirsutum*”; most other unigenes were assigned to other species, which mainly included “*Vitis vinifera*”, “*Ricinus communis*” and “*Populus trichocarpa*”. This implies that the genome of cotton may be very similar to these species and this could be a reference for a prospective cotton sequencing project.

Z263 was the offspring which derived from a cross between white fiber cotton and dark brown cotton, and Z128 was an inbred line selected from Z263 with a lighter color and better fiber quality. Therefore, the similar genetic backgrounds provided a fine model with which to study the mechanism underlying the formation of brown color in cotton fiber. We compared the whole transcriptome for each and, unexpectedly, an abundance of unigenes (20.26%) revealed significant differential expression between Z263 and Z128 (FDR≤0.001, |log_2_Ratio|≥1). This evidence demonstrates that all DEGs are relatively evenly distributed in most of the relevant metabolism pathways. Namely, the divergence of cotton fiber color and quality was the result of complex processes generated by multiple metabolic processes.

There is limited information in the literature on the molecular mechanism that underlies the formation of fiber color in cotton. Xiao et al [6] cloned five flavonoid structure genes from brown cotton fiber and found that all the cloned genes could be involved in pigmentation metabolism for brown fiber. Several studies focused on chemicals also suggested that proanthocyanidins (condensed tannins) might be the precursor of pigmentation in natural colored cotton fiber [7]–[8]. Here, almost of all the unigenes which encoded the key enzymes (CHS, CHI, LAR, ANS and ANR) of the flavonoid biosynthesis pathway were down-regulated in Z128 compared with that in Z263, implying that accumulation of proanthocyanidins in Z263 might be more than that in Z128. Zhan et al. [7]–[8] suggested that the depth of brown color might be closely related to the accumulated quantity of condensed tannins. Another unexpected finding in this study was related to the “anthocyanin biosynthesis” pathway. As a downstream metabolic pathway, it is one of the most important elements of pigment biosynthesis in plants [31]–[33]. We found that all unigenes involved in this pathway were significantly up-regulated (read in Z263 = 0) in Z128 and homologous with anthocyanin 5-O-glucosyltransferase (5GT), which could make anthocyanin more stable by modification [34]. Another recent study demonstrated that the lack of glucose at the 5 position of anthocyanin could lead to color variation in carnations [35]. This result implied that the depth of brown cotton fiber color variation might be the consequence of an interaction between oxidized tannins and glycosylated anthocyanin.

*F3H* has been thoroughly studied for decades and it is predominantly expressed during the fiber elongation stage in *G. barbadense*, a process that has no relation to pigment formation [36]. Furthermore, when the *F3H–RNAi* segment was transferred into brown fiber plants, they yielded more stunted fibers. Transgenic analysis showed that the suppression of *F3H* not only inhibited fiber elongation but also retarded fiber maturation [37]. *F3H* was suppressed in Z263 but up-regulated in Z128. This evidence suggests that *F3H* is important in fiber development. Like *F3H*, *F3*′*H* and *FLS* were very important in the pigments synthesis. Therefore, it is possible that the genes in the middle were up-regulated. According to Xiao et al [6] and Zhan et al [7], tannins could be the key chemical responsible for the brown color in cotton fiber. As shown in Fig. 5, *LAR*, *ANR*, and *ANS* were the key enzymes to accumulate the tannins. The down-regulated *CHS*, *CHI*, *LAR*, *ANR*, and *ANS* genes in the main pathway of pigment formation should be related to lighter fiber color of Z128 than that of Z263.

The pigmentation related genes not only regulated the fiber color but also influenced the fiber quality. The flavonoids are abundant and widely distributed plant secondary metabolites, and known to be an active participant in fiber development [38]. Previous studies showed that in fiber cells, the flavonoid genes were dominantly expressed in the fiber elongation stage [39]–[41]. In our study, *CHS*, *CHI*, *LAR*, *DFR*, *F3H*, *F3*′*H*, *ANR* and *ANS* showed higher expression levels at 10 DPA in Z128, thus highlighting that flavonoid metabolism represents a novel pathway with the potential for cotton fiber improvement. Our GWAS analysis of SNP in cotton germplasm indicated that the genes involved in flavonoid biosynthesis were also associated with fiber quality traits (unpublished data). Therefore, the highly expressed flavonoid gene in the fiber elongation stage in Z128 should be related to better fiber quality.

Cotton fibers are single-celled trichomes that differentiate from the ovule epidermis, including fiber initiation, elongation, secondary cell wall biosynthesis and maturation, leading to mature fibers. Ca^2+^ and ROS are two important factors involved in fiber cell growth [42]. Ca^2+^/Calmodulin (CaM) is involved in plant growth and development through interaction with ROS signaling [43]. Based on gene expression profile analysis, Ca^2+^/CaM is implicated in cotton fiber elongation. However, currently, there remains little direct evidence of the mechanism of Ca^2+^/CaM on cotton fiber development. In our study, Ca^2+^ related genes were either down-regulated or up-regulated in Z128 compared with that in Z263.

Recent literature indicates that ethylene acts as a positive regulator of root hair, apical hook, and hypocotyl development [44]–[46]. Furthermore, Shi et al. [24] found that ethylene biosynthesis was one of the most significantly up-regulated biochemical pathways during fiber elongation. Exogenously applied ethylene promoted robust fiber cell expansion, whereas its biosynthetic inhibitor L-(2-aminoeth oxyvinyl)-glycine (AVG) specifically suppressed fiber growth. The ethylene biosynthesis pathways in our data were not shown in Table 3 as the “top 10 DEGs enriched pathways in KEGG analysis”, however, a number of ethylene related genes were up-regulated in Z128 compared with that in Z263, such as Unigene61435_All, Unigene40770_All, and Unigene62869_All. This suggests that ethylene related genes may contribute to better fiber quality in Z128.

## Supporting Information

Table S1 **Classification of all-unigenes by KEGG analysis.**
(XLS)
